# Sport Participation for People with Disabilities: Exploring the Potential of Reverse Integration and Inclusion through Wheelchair Basketball

**DOI:** 10.3390/ijerph20032491

**Published:** 2023-01-30

**Authors:** Rebecca Ramsden, Rick Hayman, Paul Potrac, Florentina Johanna Hettinga

**Affiliations:** Department of Sport Exercise and Rehabilitation, Faculty of Health and Life Sciences, Northumberland Building, Northumbria University, Newcastle upon Tyne NE1 8ST, UK

**Keywords:** inclusion, reverse integration, wheelchair basketball, motivational influences, sport participation, adapted physical activity

## Abstract

Reverse integration is defined as the inclusion of able-bodied people into disability sport. For decades, there have been movements towards integrating people with a disability in mainstream society. There has been a lack of research supporting the movement of able-bodied involvement in disability sport, known as reverse integration. In this study, the real-life experiences and motivations of 11 national wheelchair basketball players (four able-bodied and seven with a disability) were explored, identifying the potential of reverse integration and what influenced the players involvement. Thematic analysis was employed using a deductive approach. The social-relational model was used as a framework to help interpret the findings. The results highlighted that health and social benefits were key factors towards prolonged engagement in wheelchair basketball, and it was reported that reverse integration led to an increased mutual understanding of the impact of (dis)ability. All participants reported positive experiences and supported able-bodied involvement, suggesting that able-bodied players play a key role and help to grow the sport locally. However, involvement of able-bodied players was not supported at international level. This challenges the concept of inclusion at higher level and whether the sport could be more inclusive. These findings could provide direction to coaches and policymakers for developing further inclusive opportunities at all levels. Further research may explore coach education programs and learning experiences of becoming an inclusive coach to ensure coaches know how to create, stimulate and coach in inclusive sport environments.

## 1. Introduction

Research has repeatedly shown several physical, mental and social benefits of physical activity for disabled people [1]. According to Sport England surveys, 1 in 5 people in England are being classed as disabled, and twice as likely to be physically inactive than those without a disability. Typical barriers for disabled people to participate in sport include a lack of awareness from those without disabilities on how to include them in sport [2], lack of opportunities for training and competition, accessible facilities causing physical barriers [3], and limited resources [4]. Negative social attitudes are sadly another common barrier in sport, causing social isolation and impact on emotional and physical wellbeing [3]. When asked in the Activity Alliance survey, two thirds (64 per cent) of disabled people would prefer to take part in sport in a mixed group of disabled and non-disabled people. Perceptions can often change when people are mixed, engaging people who are differently abled in an adaptive sport setting, for example in wheelchair basketball.

Sports participation is encouraged for disabled people to promote physical and mental wellbeing [1]. Inclusive sports can also have a positive influence towards participation engagement, hence improving physical and mental health [5]. Research on sport participation and inclusion has been well reported in the literature. However, there is a gap in research to explore players experiences of able-bodied involvement in disability sport. Reverse integration is the term used for the inclusion of able-bodied players in disability sport. Wheelchair basketball is the most popular ‘disability’ sport, and with its inclusion of mixed (dis)abilities, ages, and genders, exploring players perceptions about reverse integration can arguably provide valuable insights to further develop and improve inclusive sport environments more generally. In the British Wheelchair Basketball leagues there are currently 17,000 people playing wheelchair basketball in the UK at both regional and national level, with 200+ teams across England, Ireland, Scotland, and Wales with potential participation growth. In this case, 21% of players in the national league are said to be non-disabled, so players with and without disabilities play together. Wheelchair basketball was the first sport to introduce reverse integration, involving able-bodied players into a disability sport [6]. This became popular worldwide with more teams and more countries competing in wheelchair basketball [7]. Able-bodied involvement is said to help sustain growth of wheelchair basketball as they help to make up numbers enough for a team [8]. Without able-bodied involvement, the sport would struggle and would not be the popular sport that it is today [9]. Interestingly, at international level, able-bodied players are excluded from international opportunities as currently there is no offer for them to take part at higher level competition, due to there being enough disabled people across the country to make up a team [10]. Although able-bodied players gain the same fulfilment and challenges as the disabled players, and take the sport competitively, there is not a requirement to continue to play at international level [9].

Further, the topic of ‘ableism’ is a growing trend currently in the field of research in disability sport. Ableism is defined as the devaluation and differentiation of disability that is considered to be outside the norm in society [11]. Disabled athletes have criticized that in some sports only a small range of disabilities are included, therefore some athletes still feel excluded or misrepresented in regard to their ability [12]. At a glance, these situations look harmless but could come across as patronizing events that objectify a disabled person for the benefit of a feel-good moment [13]. Greater promotion of integrated sport where everyone has equal opportunity can help reduce ableism [14]. Future research and understanding on ableism could help to increase the diversity of disabilities represented and encourage coaches to educate themselves to be able to support the inclusion of people with disabilities into mainstream sport and/ or, varying disabilities in disability sports. Given the uniqueness of reverse integration and its inclusive qualities, more qualitative studies are needed to provide a deeper understanding of the current, lived experiences of wheelchair athletes with and without disabilities.

The social relational model of disability acknowledges the merits of both medical and social models and as a result allows us to understand the personal experiences and impairment effects in a sporting context that is in this case wheelchair basketball [15]. In this study, it will help to analyse the social construct between players in a reverse integrated setting and may be useful to highlight the dominant discourse and practices about disability in wheelchair basketball, subsequently informing approaches to provide inclusive environments in sport.

Accordingly, the aim of this paper was to explore players’ perceptions and experiences regarding reverse integration and inclusion in wheelchair basketball in order to provide a greater understanding of why people play wheelchair basketball in an integrated environment. From a social relational perspective, areas for development to enhance sport participation for all will be explored. Perspectives of players with and without disabilities will be discussed. This expands on current research, particularly on the experiences and reasons for participation in wheelchair basketball in current times and if there are any differences between able-bodied and disabled players.

Given the lack of research on both able-bodied and disabled player’s experiences of reverse integration, it was important that meaningful discussions took place with the aim to provide additional, original context to existing literature. Gaining players insights was deemed particularly important to help challenge current perceptions of disability and disability sport and share the experiences of integration of able-bodied players in wheelchair basketball. This paper has the potential to inform future practice by allowing practitioners in sport to gain better understanding and awareness of inclusion and the concept of reverse integration to enhance inclusive practices, participation engagement and opportunities offered for disabled people. 

## 2. Materials and Methods

### 2.1. Research Design

This research was underpinned by a relativist ontology and is based on the construct that no one ‘true’ reality exists and instead is ‘relative’ according to how participants feel and experience it at that given time and place [16]. With this philosophical stance, this study employed a qualitative deductive methodology to gain insight into participants’ experiences and perceptions of the central phenomena in question [17]. A qualitative design is suitable for methods involving semi-structured interviews to gain insight and knowledge of the topic in question, as well as being able to provide flexibility for transformation as ideas develop during data analysis [17].

### 2.2. Recruitment and Sampling

Upon receiving approval from the university research ethics board, participants were contacted via a local wheelchair basketball club. Thereafter, participants were selected using purposeful sampling [18]. Participants were deemed eligible to participate in this study if they were a current player in wheelchair basketball, a classifiable player according to the IWBF’s criteria, and over the age of 16 with a minimum of one year playing experience. Once the support was secured from the participants, contact and arrangements were organised to formally invite the players to participate in the study.

The final sample comprised of 11 wheelchair basketball players, males (n = 7) and females (n = 4), aged 18–41 years old, who had played in the British wheelchair basketball national league, with experience of at least one year, participated in this study. Seven players had a disability, the remaining four were able-bodied. Two of the able-bodied respondents were coaches of a wheelchair basketball club who also played on court as a player-coach in the league. All participants lived in the North East of England. In the initial contact with the participants, the purpose of the study, the nature of their participation, and the associated ethical implications were informed in accordance with the University’s ethical guidelines. All participants provided written and verbal informed consent and were given a copy of the information sheet. For confidential purposes, pseudonyms and participant numbers were used to protect the anonymity of the participants during the interview process [19].

### 2.3. Data Collection and Analysis

Semi-structured interviews were employed to encourage discussion and reflection of the participants’ views and experiences of reverse integration. A deductive approach to the semi-structured interview was taken and the interview guide was pilot tested to allow the lead researcher (first author) to practice and refine interviewing technique and ensure the questions were appropriate and easily communicated. Face-to-face interviewing was arranged with all participants at a time at a location that suited them. In this case, the sports building where they train was a suitable venue in terms of timing (before training), accessibility, and feeling comfortable in their surroundings. Participants were taken to a quiet separate room away from the noisy sports hall. This was deemed a most suitable location for the interviews as most players were there for training and were familiar with the centre, so accessing the players for interviewing was convenient. Each individual interview lasted 30–60 min. In total, 11 interviews were conducted. Elaborative and detail-oriented probes were used to elicit more in-depth responses at points of the interview, where it could discover a new inquiry or to pursue a certain topic [20], with phrases used such as “can you explain further?” “Why is that?” or “How did that make you feel?” and “Do you think it helped you?”. Each interview was audiotaped and transcribed verbatim into an electronic file.

Individual interviews were manually transcribed by the researcher and subjected to a reflexive thematic analysis [21]. Initially, the data were read and re-read to familiarise with the data. Codes were then generated based on consistent features across from the data, relevant to the main themes. The data were then collated into potential themes, generating a thematical ‘map’, ensuring the themes worked in relation to the main themes. The sub themes generated were analysed to define a meaning to the data. The final sub themes were then selected appropriately, extracting meaningful discussions relating back to the research in question. This iterative process led to a final review of the themes to assess if they resonated with and fairly reflected the data, signalling the end of the analytical process.

### 2.4. Trustworthiness and Rigour

In order to ensure qualitative rigour [22], the second author acted as a critical friend to the first author. Findings were interpreted and presented to the second author where discussions and interpretations were challenged through critical feedback. This process allowed us to identify minor changes appropriately regarding the codes and theme categorisation. The last author reviewed the end result of the analytical process ensuring we had provided meaningful data and interpretations for discussion.

## 3. Results

The findings and interpretations presented below highlight the benefits and continuity of participation in the integrated environment that wheelchair basketball offered. The data collected from current national wheelchair basketball players revealed competing interpretations of reverse integration and how it is perceived from their perspective, sharing their experiences in wheelchair basketball.

### 3.1. Reasons for Participation in Wheelchair Basketball

Participants were asked to reflect on the reasons why they participate in wheelchair basketball. Four of the players reported that wheelchair basketball had a lot of benefits to their health. All stated they had an injury or limitations from impairment and wheelchair basketball had helped them to stay active or help with recovery. Interestingly one able-bodied participant shared their experience of a small injury through rugby which prevented them from playing any running sports (see Table 1). In addition, the social benefits were also shared as an important factor towards their participation and had a positive impact on their experiences in wheelchair basketball. Most participants stated they formed new friendships, and they feel part of a small community. The social aspect of wheelchair basketball, with its mixed ability, mixed gender and mixed ages, allows people to interact and play with others. For example, one participant expressed a positive outlook of disabled people being able to play with their able-bodied siblings or parents (see Table 1). 

It is acknowledged that these benefits are important considerations towards continued participation which helps promote the benefits of inclusion and reverse integration. 

### 3.2. Perceptions on Reverse Integration

As well as highlighting the benefits of wheelchair basketball, all participants highlighted how able-bodied players are perceived in wheelchair basketball and in this case, it was shared that able-bodied players benefit the team in a supportive role but also played an important role on court. Interestingly, the able-bodied participants shared they were not aware that able-bodied people could play wheelchair basketball until they were introduced by a friend (see Table 2). The perceived importance of able-bodied players suggests a positive outcome for reverse integration. One player stated that able-bodied players help club growth and help to make up numbers enough for a team. This suggests that able-bodied involvement helps to sustain competitive opportunities in wheelchair basketball, especially for disabled players. An additional characteristic highlighted by the participants was the classification system. The classification system was viewed as a crucial tool to ensure the sport is inclusive and fair. This also determines the position and roles on court, and it was suggested that there is little difference between a player who is disabled and is classified as a 4- or 4.5-point player, compared to an able-bodied player regarding ability and role on court (see Table 2). Therefore, this challenges perceptions of players and perceived ability of players in wheelchair basketball. An interesting finding stated that wheelchair basketball could be more inclusive and a more level playing field by ensuring the height of the playing chairs are the same everyone. This has not been expressed in other literature.

### 3.3. Experiences in Wheelchair Basketball

When participants were asked about their experiences in wheelchair basketball, most able-bodied players stated that they found wheelchair basketball challenging to start with, demonstrating the physical difficulties and complexity of the skills required to play (see Table 3). They also suggested that disabled players who use a chair daily have an advantage due to their strength and coordination of being able to push themselves. Interestingly, the disabled participants did not agree arguing that able-bodied players have an advantage due to core strength, balance and being able to maneuver the chair at speed, as well as sitting in a higher playing chair. The ability to be able to perform such skill that is required in wheelchair was also seen as a seductive pull as one able-bodied participant shared, they were attracted to the athleticism and physical challenge involved (see Table 3). This eloquently challenges how disabled people are perceived and represented in sport and in wheelchair basketball.

### 3.4. Reverse Integration at International Level 

The last set of questions investigated the participants views on able-bodied participation at international level. Interestingly, most responses were not in favor of the able-bodied continuation to elite level. For example, one disabled participant expressed why they would want to play at higher level when they have their own sport (see Table 4). This appears to be the first evidence to enquire why able-bodied players would play at elite level from a disabled person’s perspective. 

As suggested previously, ‘it does not say disability basketball its wheelchair basketball’ (P3), that the wheelchairs are seen as sports equipment similar to any other sport that uses equipment. This highlights the symbolic views of wheelchairs and how they are perceived in society. This may also suggest the divide of sports and disability sports could be minimized by integrating sport competitions. For example, in the Olympics there could be potential to integrate wheelchair basketball as an Olympic sport, not just for the Paralympics. Therefore, this would promote inclusivity and involve able players at international level.

This finding has not been well discussed in previous studies and therefore could be room for further research development regarding the status of wheelchair basketball at a higher level of competition. All participants were asked the same question. However, responses were very mixed from both able-bodied and disabled participants. 

## 4. Discussion

This study explored the potential of reverse integration and inclusion through wheelchair basketball deductively. The social-relational model [23] was used to provide context to the findings, exploring individual experiences and perceptions of reverse integration. According to the social relational model, disability is caused by social barriers not by impairments [24]. People without disabilities are consequently viewed as in an advantaged position. A social relational approach perhaps opens up the possibility of further understanding (dis) ability in wheelchair basketball, taking into account perceptions and experiences of both able-bodied and disabled players. This also has the potential to challenge current perceptions of people who play wheelchair sport but also, it could tackle some of the barriers that people may face and thereby help to develop an inclusive approach towards disability and disability sport. 

### 4.1. Reasons for Participation in Wheelchair Basketball

One of the aims of this study was to explore the motivations of players who play wheelchair basketball, identifying how they got involved and why they play the sport. Firstly, the results highlighted how players were involved in wheelchair basketball. The majority of disabled participants stated they were introduced to wheelchair basketball through post-injury rehabilitation as part of their recovery, with health benefits as an important motivation. Most disabled players acquired their disability through injury, while two players shared, they were born with their disability. Interestingly, the able-bodied participants also shared their experiences of injury, causing them to discontinue playing ‘running’ sports. However, it was stated they were unaware that able-bodied players could play wheelchair basketball until they were introduced to the sport by someone they knew. 

A social relational understanding helped to highlight some of the benefits of wheelchair basketball and demonstrated why players continued to play. For example, on the field, disability does not matter, and it is down to the individual and putting in the work. This helped to recognise the social construction of disability and personal experiences, including the lived experiences [24]. The results also highlight the perceived benefits of playing wheelchair basketball, such as physical and mental benefits [25]. The social element of wheelchair basketball was said to have a positive impact on all participants and helped them with their mental health. Social interaction and a sense of belonging, being part of a community, were outlined and contributed to their long-term engagement in the sport, enabling them to stay active. This demonstrates the importance of inclusion and the social opportunity between disabled and non-disabled people who potentially would not meet outside of wheelchair basketball [26]. The benefits of interaction for able-bodied players relate to learning to understand more about those who live with a disability, becoming more disability aware and realising the physical capabilities involved in wheelchair basketball [26]. Referring back to the social-relational model [24], this demonstrates a positive outcome of interaction between people and understanding disablism and impairment. The impairment effect of the social-relation model was used to refer to the understanding of restrictions of activity in the lives of people with impairment and in this case, able-bodied players gained a better understanding of disability through social interaction and what using a wheelchair is similar to [27].

The social environment has recently been explored as a supportive factor in the context of self-regulated learning of exercise [28], and in this case, long term engagement is influenced by the social environment of the players, which indeed seems an important factor for all players to remain engaged in the sport. There was also a sense of appeal from most able-bodied players in relation to the new challenge of being able to learn a ‘different’ sport. This links in well with the study of self-regulation in terms of learning new skills for intrinsic reasons (positive emotions, social interaction, and sport-related progression), which play a role towards long-term engagement [28]. This discussion is also expressed by Hutzler (2016) who also found that able-bodied players gained positive experiences from the sport and eventually taking up the sport long term as a ‘serious leisure’ activity involving more competition. This is an interesting discussion on behaviours and self-perceptions of playing wheelchair basketball as an able-bodied player as we now turn the focus on how people are perceived, including able-bodied people, playing wheelchair sports. Through a social relational lens, this reflects positive relational outcomes regarding social interaction and relationships formed, providing a meaning towards prolonged engagement in the sport [24].

### 4.2. Experiences of Reverse Integration

Our second aim was to explore the experiences of the reverse integrated environment. This was to gain a better understanding from the players perspective of their experiences in wheelchair basketball which may contribute to providing further knowledge of inclusive practices. The results demonstrated that there are no differences between players on court other than classification points in relation to ability or being treated differently [8]. The acknowledgement that all players are viewed the same on court reflects a positive, inclusive environment, thus highlighting the contribution of the social relational model of disability in recognising the ability not disability. Naturally, it was stated from all disabled participants that those who are ‘more able’ are said to have an advantage in terms of being able to use more of their body to carry out certain skills, as well as having strength and balance. All able-bodied participants stated that they do not have any advantage, and they found it very challenging to carry out the dual-task skills required in wheelchair basketball. Ableism and the presumptions that able-bodied people are more able in wheelchair basketball are challenged here. In this case, neither able-bodied nor disabled players are favoured in wheelchair basketball and therefore it is an equal game between players. Perceptions of players are queried here, interestingly from both able-bodied and disabled participants stating they do not have an advantage, which suggests it comes down to the commitment and learning of skill that influences their playing ‘ability’ [9]. Therefore, the impact towards the societal attitudes of people in wheelchair basketball have been challenged, and has the potential to compound these attitudinal conflicts in sport and how players perceive themselves and others [29].

Further to this discussion, an original aspect of this study relates to enquiring why able-bodied players would play a wheelchair sport, especially at international level, when they do not have an impairment which requires a chair daily. This led us to dive more into able-bodied involvement at international level, for example at the Paralympics. This perception has not been well discussed in other studies, identifying the need for thought when approaching ‘inclusion’ at competitive level and what approach would be needed to take to make it inclusive at higher level not just club level. At club level, it is expressed that everyone is treated equally [8]. However at a competitive level there is a risk of people feeling excluded [12]. Interestingly in this case, it is now the able-bodied players who feel excluded from international opportunity, what could be known as reverse discrimination [6] and provides additional insights into ‘ableism’ in that the players who have an ‘eligible’ disability are favoured more [12]. People with disabilities are unfortunately being excluded from international wheelchair basketball. Those who meet the performance requirements and within the classification system are selected accordingly [30]. This is very common in the Paralympics where players are re-classified based on their eligible disability [31], meaning some disabled players do not have an eligible disability and therefore, are excluded in the chance to represent their country [32].

While the players from our interviews had recognised the benefits of wheelchair basketball, the importance of classification was set to be essential to keeping the sport inclusive. The classification system has been developed as a crucial tool to ensure the sport is inclusive [33], allowing players of different ability to play in the same competition together. Classification is based on the extent in which an impairment impacts on the sport performance. Once eligibility is confirmed players are assigned a classification from 1.0–4.5 points based on their functional ability. Non-disabled players and players who do not have an eligible impairment are assigned the highest classification point of 5.0 [34]. Some disabled participants expressed that even though able-bodied players are vital for the team, it would be unfair to have more than one or two able-bodied players on court at any one time, which is why the point system is in place which caps the total sum of classifications of players on court. 

Positive experiences of engagement in wheelchair basketball were shared from all participants and provided an understanding and awareness of (dis) ability and roles within the team. Wheelchair basketball seems a nice example of an integrated sport that can offer us the key components and knowledge, such as using a reverse integrated approach, for developing more inclusive sport environments in other sports. However, there is a need for more promotion and research on this, especially at club level, as now most research on wheelchair basketball only explores wheelchair basketball at competitive level with a strong performance focus [35].

Governing structures, including national governing bodies, play an important role in making sport accessible and inclusive at all levels. This study has highlighted the benefits and positive outcomes of reverse integration and its inclusivity at grass roots, and order to sustain disability sport opportunities in local areas. Wheelchair basketball has the potential to promote long term engagement offering perceived health benefits, creating a sense of belonging, and facilitating mutual understanding through social interaction. The study findings contribute towards understanding disability in a social relational model of disability, and to the understanding of the social construct of an inclusive sport and has challenged the views of ableism, especially at international level. This study has the potential to inform further studies of the potential of reverse integration and inclusivity in sport in order to sustain sport participation. 

### 4.3. Practical Implications, Limitations, and Future Research

There are several practical implications that may inform future practitioners in all levels of sport. Practitioners working with people who have disabilities have the potential to facilitate and sustain inclusive opportunities in their sport offer. Practitioners can promote the benefits of inclusion, particularly reverse integration, in order to enhance sport participation and engagement in sport and disability sport. Practitioners should continually engage in developmental learning opportunities to increase their knowledge and understanding of disabilities so that they are able to offer inclusive sport.

Access to participants when the data collection was taking place served as a limitation to this study as the basketball season had ended, therefore has limited transferability and generalization regarding data interpretations based on participants being from the same team. Additionally, limited research on players’ experiences of reverse integration and motivations in participation in wheelchair basketball in other literature creates a gap in the discussion to compare against other findings. Future research should explore coaches’ experiences and learning pathways of becoming a disability coach. Further research could also investigate coach educational needs and explore potential learning avenues to develop knowledge and good practice regarding inclusion in sport.

## 5. Conclusions

This paper begins to shed light on the potential of wheelchair basketball in creating more inclusive sport opportunities. This study highlights the health benefits and social benefits as being the main determinants to participation from players with and without disabilities. These considerations can help drive the understanding of why people play wheelchair basketball as well as demonstrating the benefits of including able-bodied players into a disability sport. The playing experiences of able-bodied and disabled players were shared. From a social relational perspective, findings provided greater mutual understanding and awareness of (dis) abilities involved in wheelchair basketball with the ontology that challenges the perceptions and representation of disabled people in sport. The findings from this study can inform future practitioners interested in offering inclusive sport environments involving able-bodied and disabled people. Considerations of how coaches learn to become a disability sports coach could provide greater understanding to sustain inclusive sport participation for all. Moreso, there is a need for more inclusive coaches as this would help to minimize some of the barriers that people face regarding participation in sports. More research is required across the disability coach development pathway and how coaches can contribute to increase participation and in offering inclusive opportunities.

## Figures and Tables

**Table 1:** Benefits of wheelchair basketball.

Sub Themes	Results
Health Benefits	‘I joined wheelchair basketball through Help for Heroes during my recovery from an injury in the army’ (P8).
	‘I have lumbar spine disease, nerve damage and I suffer from PTSD, I play to get me active and help with my health’ (P9).
	‘I find playing wheelchair basketball is good for my mental health’ (P3).
	‘I used to play rugby but then I had gained a few small injuries which prevented me from playing any kind of running sports, then I found wheelchair basketball’ (P4).
Social Benefits	‘We have a saying in my house that wheelchair basketball is like a dating agency. I met my girlfriend through wheelchair basketball’ (P6).
	‘I think it’s a fantastic thing to have so everybody can play, we’ve got junior clubs here and there are a lot of brothers and sisters who can therefore play and mums and dads and everybody can play together so it’s nice that its inclusive, but it’s done in the right way’ (P7).
	‘I have actually made life long friends through wheelchair basketball who I would never have met in day-to-day life’ (P9).

**Table 2:** Players’ perceptions of able-bodied involvement in wheelchair basketball.

Sub Themes	Results
How able-bodied players are perceived	‘I didn’t know able-bodied people could play until I went along with my partner, and I got asked if I wanted to join in’ (P3)
	‘They bring a lot to the team, they do all the dirty work and because of their height and their core strengths and the use of their legs, they have more stability, so they’re the ones who are fighting for the ball from up high and get in the dirtiness’ (P8),
	‘if we didn’t have the abled bodied players then we wouldn’t have enough for a team’ (P2)
	‘On the court, they are not much more “able” than a 4.5 disabled player’ (P6).
	‘Obviously, the classifications are about disability, but it does not say disability basketball its wheelchair basketball so add the equipment in and everybody is the same’ (P3).
	‘Just because it is open to everyone doesn’t mean that able-bodied players can just come in and get in a higher chair with bigger wheels and be better on court. I mean there’s not much difference between some players, like your 4.5 players and 5-point players but when you’re playing against other teams who have like 3 or 4 ‘tall more able’ players, who are obviously sitting higher, us ‘small’ players have no chance of defending them. I think that to me would make it a more level playing field, everyone should be in small chairs’ (P11).

**Table 3:** Players experiences in wheelchair basketball.

Sub Themes	Results
Perceived ‘ability’ in wheelchair basketball	‘Being able to move a wheelchair, I struggled for two years to be able to do the skills that are required and then I saw people who use a wheelchair daily and they were so much better than me to start with and I found it frustrating. I faced many problems playing it’ (P7).
	‘Even though I am wheelchair bound I found learning to use the sports chair very different and challenging to co-ordinate everything like pushing and dribbling the ball at the same time, especially when I don’t have any balance or core strength anyway. I think the able-bodied players have an advantage when it comes to that. We have to adapt more with our disability, our parts of the body which can’t co-ordinate as we would like to in that sense’ (P11).
	Responses from most disabled participants revealed that it is not whether you are disabled or not, or whether you use a wheelchair on day-to-day basis, it is down to the individual and whether they are willing to put in the hard work and training that is required to learn these skills. ‘It comes down to the skill and time that you are willing to put in’ (P8).
	‘It’s something you look at and it’s so skilled, and it is wanting to be able to do all those things, and when you see it at the Paralympics and the players are doing all these plays and I think it would be really cool to be canny as good as that’ (P3).

**Table 4:** Players’ views on able-bodied participation at international level.

Sub Themes	Results
Reverse integration at international level	‘I don’t think they should be included at international level; they have their own Olympics. They were only allowed to play at club level to make up numbers and help grow the sport, but at elite level they aren’t needed, there’s enough players across the UK to make up the GB team. Why would they want to play at elite level anyway? I know the Invictus Games allow it, but they have a different system in place, but it works I guess’ (P11).
	Since wheelchair basketball first began, there has been a growth of countries set on board with the inclusion of able-bodied athletes in the sport. Although the inclusion of able-bodied players is important for participant numbers at local clubs, it appears that when it comes to competition at a higher-level, able-bodied players are treated differently. ‘I don’t see the need for it, there are enough disabled people to form a team at international level, we don’t need able-bodied players’ (P5).
	‘It does not say disability basketball its wheelchair basketball’ (P3).

## Data Availability

The data presented in this study are available upon request.
